# Modifiable factors affecting renal preservation in type I glycogen storage disease after liver transplantation: a single-center propensity-match cohort study

**DOI:** 10.1186/s13023-021-02026-6

**Published:** 2021-10-11

**Authors:** Yi-Chia Chan, Kai-Min Liu, Chao-Long Chen, Aldwin D. Ong, Chih-Che Lin, Chee-Chien Yong, Pei-Chun Tsai, Liang-Suei Lu, Jer-Yuarn Wu

**Affiliations:** 1 Liver Transplantation Center Department of Surgery, Kaohsiung Chang Gung Memorial Hospital, Chang Gung University College of Medicine, 123 Ta-Pei Road, Niao-Sung, Kaohsiung, 83303 Taiwan; 2 Institute of Biomedical Sciences, Academia Sinica, 128 Academia Road, Section 2, Nankang, Taipei, 11529 Taiwan

**Keywords:** Kidney, Renal function, Glycogen storage disease, Liver transplantation

## Abstract

**Background and aims:**

Glycogen storage disease type I (GSD-I) is an autosomal recessive disorder of carbohydrate metabolism, resulting in limited production of glucose and excessive glycogen storage in the liver and kidneys. These patients are characterized by life-threatening hypoglycemia, metabolic derangements, hepatomegaly, chronic kidney disease, and failure to thrive. Liver transplantation (LT) has been performed for poor metabolic control and delayed growth. However, renal outcome was diverse in pediatric GSD patients after LT. The aim of this study was to investigate the long-term outcome of renal function in pediatric GSD-I patients after living donor LT (LDLT), and to identify modifiable variables that potentially permits LT to confer native renal preservation.

**Methods:**

The study included eight GSD-Ia and one GSD-Ib children with a median age of 9.0 (range 4.2–15.7) years at the time of LT. Using propensity score matching, 20 children with biliary atresia (BA) receiving LT were selected as the control group by matching for age, sex, pre-operative serum creatinine (SCr) and pediatric end-stage liver disease (PELD) score. Renal function was evaluated based on the SCr, estimated glomerular filtration rate (eGFR), microalbuminuria, and morphological changes in the kidneys. Comparability in long-term renal outcome in terms of anatomic and functional parameters will help to identify pre-LT factors of GSD-I that affect renal prognosis.

**Results:**

The clinical and biochemical characteristics of the GSD and BA groups were similar, including immunosuppressive regimens and duration of follow-up (median 15 years) after LT. Overall, renal function, including eGFR and microalbuminuria was comparable in the GSD-I and BA groups (median eGFR: 111 vs. 123 ml/min/1.73m^2^, *P* = 0.268; median urine microalbuminuria to creatinine ratio: 16.0 vs. 7.2 mg/g, *P* = 0.099, respectively) after LT. However, in the subgroups of the GSD cohort, patients starting cornstarch therapy at an older age (≥ 6-year-old) before transplantation demonstrated a worse renal outcome in terms of eGFR change over years (*P* < 0.001). In addition, the enlarged kidney in GSD-I returned to within normal range after LT.

**Conclusions:**

Post-LT renal function was well-preserved in most GSD-I patients. Early initiation of cornstarch therapy before preschool age, followed by LT, achieved a good renal prognosis.

**Supplementary Information:**

The online version contains supplementary material available at 10.1186/s13023-021-02026-6.

## Introduction

Glycogen storage diseases (GSD) are inborn errors of metabolism with abnormal storage or utilization of glycogen, caused by enzyme deficiency, affecting glycogen synthesis or breakdown, or from mutations in proteins regulating glycogen metabolism [1]. GSD type I (GSD-I) consists of two major subtypes, GSD type Ia (GSD-Ia) and type Ib (GSD-Ib), caused by mutations in the G6PC and SLC37A4 genes, respectively. In glycogenolysis, glucose 6-phosphate (G6P) is transported from the cytosol into the lumen of the endoplasmic reticulum (ER) via the enzyme, glucose 6-phosphate translocase (G6PT, encoded by the SLC37A4 gene). G6P is hydrolyzed into free glucose by the enzyme, glucose-6-phosphatase (G6Pase, encoded by the G6PC gene) in the ER. Deficiency in either enzyme function markedly reduces production of free glucose and result in accumulation of glycogen and excessive fat in the liver, kidneys, and intestinal mucosa, which subsequently results in hypoglycemia, lactic acidosis, hyperuricemia, and hyperlipidemia. Clinical manifestations typically present within the first year of life, with features that often include growth retardation, hepatomegaly, fatty liver, neutropenia (GSD-Ib), and renal dysfunction, secondary to nephrocalcinosis and/or glomerulosclerosis [2–4].

Development of renal dysfunction in GSD-I cases was first reported by Chen et al. in 1988 [2]. GSD nephropathy is a frequently reported complication, probably primary due to enzyme deficiency in the kidneys or secondary to the abnormal metabolic environment resulting from enzyme deficiency in the liver [5]. However, if metabolic derangement is controlled, the incidence of kidney damage can be lower [6]. Furthermore, a study indicated that GSD-I patients with early dietary treatment had less proteinuria than those with late treatment, suggesting that correction of metabolic derangement early in life may prevent or slow the progression of renal disease [7].

The principle of treatment for GSD is to maintain normoglycemia by continuous or frequent nutrition therapy with glucose, meals, or cornstarch day and night, affecting the quality of life for patients and their parents [4, 8]. Notably, although optimization of serum lactate, lipid, and uric acid levels with continuous glucose therapy may delay or prevent the occurrence of associated complications [9, 10], the development of liver adenoma is not uncommon in well-controlled GSD-I patients with the potential for tumor rupture, hemorrhage, and malignant transformation to hepatocellular carcinoma [11]. For these reasons, liver transplantation (LT) have also been known to become indicated in some patients [4, 12–14].

In contrast to glucose therapy, LT provides a healthy liver graft that not only corrects the genetically acquired error of metabolism but also mitigates the risk of developing adenoma growth or liver cirrhosis [12, 14]. Although LT corrects glucose homeostasis and metabolic derangement, some GSD patients receiving LT progress to renal insufficiency or end-stage renal disease (ESRD) [7, 15]. Whether the development of CKD in GSD-I patients after LT is attributable to the nature of GSD progression in the kidneys itself or secondary to LT surgery or immunosuppression therapy remains unclear. Therefore, this study aimed to delineate whether the development of renal dysfunction after LT is related to disease pathophysiology, and investigate the factors affecting long-term outcome of renal function in GSD-I patients after LT.

## Patients and methods

### Study population and design

Kaohsiung Chang Gung Memorial Hospital, Taiwan, maintains a longitudinal database of primarily living donor liver transplantation (LDLT) recipients and records all demographic, pre-operative, peri-operative, pathological, and follow-up information. A total of 339 children underwent pediatric LDLT in our institution from June 1994 to December 2019. There were 11 GSD-I cases, however, two patients died before 2019 (one due to pancreatitis and the other one from chronic rejection), with nine surviving GSD-I patients undergoing regular surveillance at an out-patient basis. For all GSD recipients, liver and renal function, metabolic biochemistry, growth development, and gene expression of GSD mutation were studied. To minimize bias associated with complications related to the LDLT procedures and long-term immunosuppression which may affect renal function, we enrolled biliary atresia (BA) patients receiving LDLT as control. To adjust for bias due to variations in baseline characteristics, we applied propensity score -matching analysis in the biliary BA groups in our center for comparison with the GSD groups. Propensity scores were calculated by logistic regression, adjusting for the following preoperative covariates: age, sex, preoperative serum creatinine (Scr) level, and pediatric end-stage liver disease (PELD) score. A 1:2 match was performed using the nearest-neighbor matching method. Patients’ characteristics have been summarized in Table 1.

**Table 1:** Clinical and biochemical characteristics of the study population

	GSD group (n = 9)	BA group (n = 20)	*P* value
Age at LT (years)	9.0 (4.2–15.7)	4.8 (0.7–17)	0.144
Female sex, n (%)	6 (67%)	10 (50.0%)	0.412
Body weight (kg)	20.6 (12.0–57.3)	16.8 (8.2–63.8)	0.437
Body height (cm)	113 (92–151)	105 (76–170)	0.571
PELD score	0 (− 4 to 6.0)	− 0.5 (− 10 to 16)	1.000
Preoperative laboratory variables			
Albumin (g/dL)	4.2 (3.1–5.3)	3.8 (1.9–4.5)	0.013
Total bilirubin (mg/dL)	0.5 (0.1–1.0)	1.9 (0.3–17.1)	0.001
Creatinine (mg/dL)	0.5 (0.3–0.9)	0.4 (0.1–0.6)	0.187
eGFR (ml/min/1.73 m^2^)	104 (58–135)	125 (76–234)	0.238
Perioperative variables			
GRWR	1.63 (1.27–2.69)	1.9 (1.0–3.8)	0.976
Blood loss (ml)	120 (20–1480)	130 (35–460)	0.698
Adenoma	4 (44%)	0 (0%)	0.002
Post-LT AKI, n (%)	4 (23.5%)	13 (76.5%)	0.422
Immunosuppressive regimen			
Cyclosporin initially, n (%)	9 (100%)	20 (100%)	1.000
Tacrolimus conversion, n (%)	2 (22%)	5 (25%)	0.874
mTOR conversion, n (%)	3 (33%)	0 (0%)	0.044
Rejection, n (%)	2 (22%)	4 (20%)	0.893
De novo HTN, n (%)	2 (22%)	0 (0%)	0.032
Postoperative variables			
Total bilirubin (mg/dL)	0.8 (0.7–1.1)	1.1 (0.4–1.8)	0.177
Creatinine (mg/dL)	0.7 (0.5–3.1)	0.7 (0.5–1.0)	0.813
eGFR (ml/min/1.73 m^2^)	111 (18–175)	123 (73–174)	0.268
eGFR < 60 (ml/min/1.73m^2^), n (%)	2 (22%)	0 (0%)	0.032
Albuminuria			
Microalbuminuria (ACR, mg/g)	16.0 (5.6–1047)	7.2 (2.5–68.6)	0.099
Macroalbuminuria (ACR ≥ 300 mg/g)	2 (22%)	0 (0%)	0.089
Follow up (years)	15.4 (4.1–22.4)	15.4 (4.7–24.3)	0.637

Values are expressed as median (range) or number (percentage)

ACR—urine microalbuminuria to creatinine ratio; AKI—acute kidney injury; BA—biliary atresia; CKD—chronic kidney disease, defined by KDIGO 2012 guideline; eGFR—estimated glomerular filtration rate; GRWR—graft to recipient weight ratio; GSD—glycogen storage disease; HTN—hypertension; LT—liver transplantation; mTOR—mammalian target of rapamycin; PELD score—pediatric end-stage liver disease score

### Inclusion and exclusion criteria

In this retrospective study, all living GSD-I patients (n = 9) who underwent LDLT due to poor response to medical treatment were included; patients who died before 2019 have been excluded. All GSD-I (n = 9) and selected BA (n = 20) recipients consented to participate in the study with no subsequent dropouts from loss of follow-up. The study has been approved by the Institutional Review Board (IRB no. 201800281B0C101) and enrolled patients were provided with written informed consent.

### Definitions and formulae

The estimated glomerular filtration rate (eGFR) was calculated using the updated Schwartz formula for children (1–18 years old) and shifted to Modification of Diet in Renal Disease (MDRD) formula upon reaching adulthood [16, 17]. Microalbuminuria has been defined as an abnormal increase in the albumin excretion rate within the specific range of 30–299 mg of albumin per g of creatinine (microalbumin to creatinine ratio, ACR); macroalbuminuria has been defined as an abnormal increase in the albumin excretion rate of 300 mg albumin per g creatinine or higher [18]. We adopted the definitions of chronic kidney disease (CKD) used in the Kidney Disease: Improving Global Outcome (KDIGO) 2012 guideline [19], where it has been defined as kidney damage or MDRD with an eGFR < 60 ml/min/1.73m^2^ lasting more than three months, irrespective of etiology.

Acute kidney injury (AKI) in children was defined by KDIGO criteria and classified into three stages by the increase in Scr [20]: stage 1 refers to an increase in creatinine ≥ 0.3 mg/dL within 48 h or 1.5–1.9 times baseline within 7 days, stage 2 refers to an increase in creatinine of 2.0–2.9 times baseline within 7 days, stage 3 refers to an increase in creatinine of > 3.0 times baseline or ≥ 4.0 mg/dL, with an acute increase of at least 0.5 mg/dL, or the need for renal replacement therapy within 7 days. Urine output was not recorded in this study and was not included in the classification of AKI. Post-LT AKI is based on changes in SCr from baseline creatinine within 7 days postoperatively [21].

Ultrasound examinations were used to determine the kidney length, measured as the maximum pole-to-pole distance along the longitudinal plane in centimeters (cm). Bilateral kidney length measured were expressed in z score as corresponding to the normal distribution within the same age group [22, 23]. Nephromegaly is defined as falling outside 2 standard deviations (SDs) above the mean size by age group.

### Data collection

Demographic and operative variables included age, sex, and underlying etiology of liver disease, in addition to the coincident diagnosis of hepatic adenoma on histologic examination of the explanted liver. Preoperative variables measured include PELD score, body height (BH), body weight (BW), body mass index (BMI), serum albumin, aspartate transaminase (AST), total bilirubin (Bil), SCr, eGFR, fasting glucose, uric acid (UA), total cholesterol (Chol), and triglyceride (TG) levels. Perioperative variables include intraoperative blood loss and graft-to-recipient weight ratio (GRWR). Postoperative data included de novo hypertension (HTN), AKI, growth development, as well as liver and renal functions, being continuously monitored every three to six months in the out-patient-clinic.

The primary outcome in this study was the dynamic long-term changes of renal function post-LT as evaluated by serum Cr, eGFR, presence of albuminuria and changes in sonographic kidney length. Secondary outcomes included the GSD genetic study of Taiwanese, correction of metabolic disturbance, and recipients’ age-adjusted growth parameters (height, weight, and BMI) after LDLT.

### Pre-operative assessment, decision-making, and LDLT procedure

The pre-operative assessment included psychological examination and radiological assessment of the hepatic vasculo-biliary anatomy of both the donor and recipient with liver computed tomography (CT) angiography, magnetic resonance imaging and echography. The decision to proceed to LDLT was made in weekly multidisciplinary meetings.

### Immunosuppression therapy

All LDLT patients in the cohort received an initial standard triple immunosuppression regimen of cyclosporin A (CyA), prednisolone, and azathioprine. Target serum CyA levels were gradually decreased from 1000 to 100–150 ng/ml within the first month after LDLT. Prednisolone was weaned off over one to two years, and azathioprine was discontinued at one-year post-LDLT. When rejection occurred or pediatric patients transitioned into adulthood, CyA was switched to orally taken tacrolimus (FK) as an alternative calcineurin inhibitor (CNI). In patients with elevated SCr during follow-up, mammalian target of rapamycin (mTOR) inhibitor was given in place of CNIs to preserve renal function.

### DNA extraction and Sanger sequencing

The genomic DNA of all nine GSD-I patients were extracted from whole blood using the Gentra Puregene Blood Kit (QIAGEN) followed by manufacturer's protocol. Primers were designed for the exon sequencing of *G6PC* and *SLC37A4* gene of the patients’ DNA (Additional file 1: Table S1). Polymerase chain reaction (PCR) was performed using the T100 Thermal Cycler (Bio-Rad, Hercules, CA, USA) with Fast-Run™ Advanced Taq Master Mix (Protech, Taiwan). PCR products were then sequenced using an ABI3730 DNA sequencer (Applied Biosystems, Foster City, CA, USA).

### Statistical analysis

Data was collected and analyzed using IBM SPSS version 20 statistical software (IBM corporation, Armonk, NY). Qualitative variables in both GSD and BA groups were expressed as frequency of events and cumulative incidence (in percentage) and compared using the chi-squared test. Quantitative variables were expressed by their median with range and compared using the Mann–Whitney *U* test. Pre-LT and post-LT data were compared using the Wilcoxon signed-rank test. The annual change in the eGFR (mean ± 2 SDs) were grouped and calculated by generalized estimating equation. The association between age of starting cornstarch and microalbuminuria was performed with Spearman’s test. A *P* value < 0.05 was considered statistically significant.

## Results

### Demographic data of the GSD-I and BA groups

The study included nine GSD-I patients. Using the propensity score matching model, 20 BA patients were selected for comparison (Table 1). The preoperative age, sex, height, weight, PELD score, SCr level, and eGFR were comparable between both groups. Hyperbilirubinemia (serum total bilirubin 0.5 vs. 1.9 mg/dl in the GSD and BA groups, respectively; *P* = 0.001) and hypoalbuminemia (serum albumin 4.2 vs. 3.8 g/dL, respectively; *P* = 0.013) can be explained by the underlying pathophysiologic course of BA, wherein the main clinical outcome in patients include obstructive jaundice and subsequent biliary cirrhosis. Intraoperative blood loss and GRWR between the two groups were similar. The histopathologic finding of adenoma exclusive to the GSD-I group, more specifically, all included patients in the group who underwent LT beyond the age of 9 (age range: 9.5–15.7 years, Table 3). The median follow-up time duration was over 15 years (15.4 years; range 4.1–24.3 years), and the postoperative liver function, SCr level, and eGFR were comparable between the two groups.

CyA was switched to FK in 22% of GSD-I and 25% of BA patients for convenience of drug intake (*P* = 0.874). However, 33% of GSD-I patients were switched to mTOR inhibitor because of elevated SCr (n = 2, No. 18 and 28) and cyclosporin related post-transplant lymphoproliferative disease (n = 1, No. 1344). The rejection rate was comparable between the two groups (22% vs. 20% in the GSD and BA groups, respectively; *P* = 0.893, Table 1).

### Mutation analysis in GSD-I patients

All exons and splicing sites of the *G6PC* and *SLC37A4* gene were screened in the nine GSD-I patients. Homozygous or compound heterozygous mutations including c.648G > T (also known as c.727G > T), p.R83H, p.H119L and p.I341N in the *G6PC* gene were found in eight of the GSD-I patients (Table 2). All of these are known disease mutations of GSD-Ia. A p.R300C mutation was discovered in the *SLC37A4* gene in case no.27 (Table 2). However, no other mutations in other exons and splicing sites were identified. In addition, the patient with GSD-Ib who was pre-operatively neutropenic demonstrated an increase in absolute neutrophil count from 1276/mm^3^ pre-LT to 1996/mm^3^ post-LDLT.

**Table 2:** The development of estimated glomerular filtration rate (eGFR, ml/min/1.73 m^2^) over the years

LT No	GSD type	Gene	Mutation	Pre GFR	1y GFR	2y GFR	3y GFR	4y GFR	5y GFR	6y GFR	7y GFR	8y GFR	9y GFR	10y GFR	11y GFR	12y GFR	13y GFR	14y GFR	15y GFR	16y GFR	17y GFR	18y GFR	19y GFR	20y GFR	21y GFR	22y GFR
18	Ia	G6PC	c.727G-> T/c.727G-> T	58	73	76	63	106	86	85	60	50*	28^#^	22	14	11	5	4	3	4^@^	NA	NA	NA	NA	19	17
27	Ib	SLC37A4	p.R300C/undetected	127	104	64	93	125	102	88	94	98	86	116	121	107	104	130	140	167	142	132	153	139	122	116
28	Ia	G6PC	p.I341N/c.727G-> T	93	73	54	51	66	72	67^#^	46*	59	76	85	74	73	63	68	62	62	63	65	58	55	56	53
164	Ia	G6PC	p.R83H/c.727G-> T	135	158	85	77	82	128	126	117	130	132	103	122	107	112	90	90	111						
215	Ia	G6PC	p.H119L/c.727G-> T	117	55	107	94	112	126	128	179	150	154	196	175	155	168	134	144							
235	Ia	G6PC	p.R83H/p.I341N	104	83	76	102	114	110	109	119	115	122	98	104	107	106	98	97							
244	Ia	G6PC	c.727G-> T/c.727G-> T	98	86	79	99	108	100	94	81	82	92	119	117	130	117	124	108							
281	Ia	G6PC	c.727G-> T/c.727G- > T	85	116	87	138	154	161	182	152	132	162	167	214	150	146	175								
1344	Ia	G6PC	p.R83H/c.727G-> T	130	146*	143	158	128																		

^*^Time point of shifting calcineurin inhibitor (CNI) to mammalian target of rapamycin (mTOR) inhibitor

^#^Time point of occurrence of de novo hypertension

^@^Time point of cadaveric renal transplantation

### Liver and metabolic function and growth development in GSD patients

**Fig. 1:**
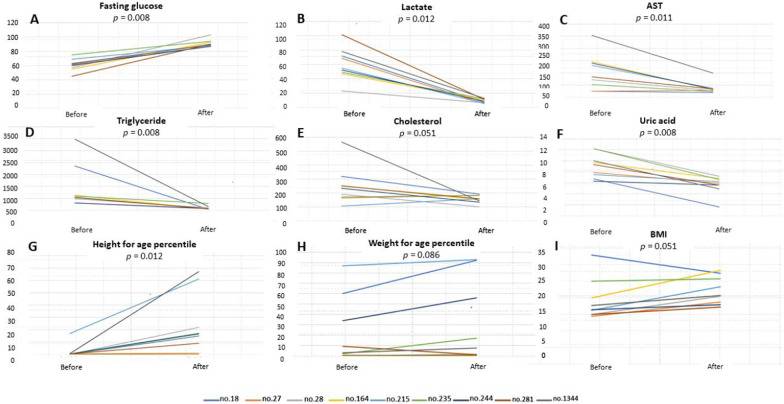
Clinical and biochemical parameters in 9 GSD patients before and after LT. **a** Changes in fasting glucose, **b** lactate, **c** aspartate transaminase (AST), **d** triglyceride, **e** cholesterol, **f** uric acid, **g** height for age percentile, **h** weight for age percentile, **i** body mass index (BMI). *P* indicates the difference between values before and after LDLT

The line graphs showing the pre-to post-LT linear changes of measured variables including fasting glucose (*P* = 0.008), lactate (*P* = 0.012), AST (*P* = 0.011), TG (*P* = 0.008), Chol (*P* = 0.051), UA (*P* = 0.008), and growth parameters by percentile according to age group in terms of BH (*P* = 0.012), BW (*P* = 0.086), and BMI (*P* = 0.051) are visualized in Fig. 1. Fasting glucose, lactate, AST, TG, UA, and growth parameters showed overall significant improvement after LDLT in GSD-I patients; Chol and BMI showed borderline improvement.

### Biochemical renal function: SCr and eGFR

Although the overall postoperative eGFR was comparable (111 vs. 123 ml/min/1.73 m^2^, *P* = 0.268; Table 1) between GSD-I and BA cohorts, two GSD-I patients (No. 18 and 28) had eGFR < 60 ml/min/1.73 m^2^ that persisted for more than three months (22% vs. 0%; *P* = 0.032). GSD-I patient No. 18 underwent renal transplantation 16 years after LT for progressive renal deterioration; conversely, patient No. 28 demonstrated improved renal function after adjustments in the immunosuppression therapy (Table 2). Both patients had concurrent de novo HTN at the time of CKD diagnosis, which they were treated with anti-hypertensive drugs. The annual eGFR of all GSD-I recipients after LDLT is shown in Table 2. Before the end of the study period, seven GSD-I patients (77.8%) were able to maintain eGFR above 90 ml/min/1.73 m^2^.

**Fig. 2:**
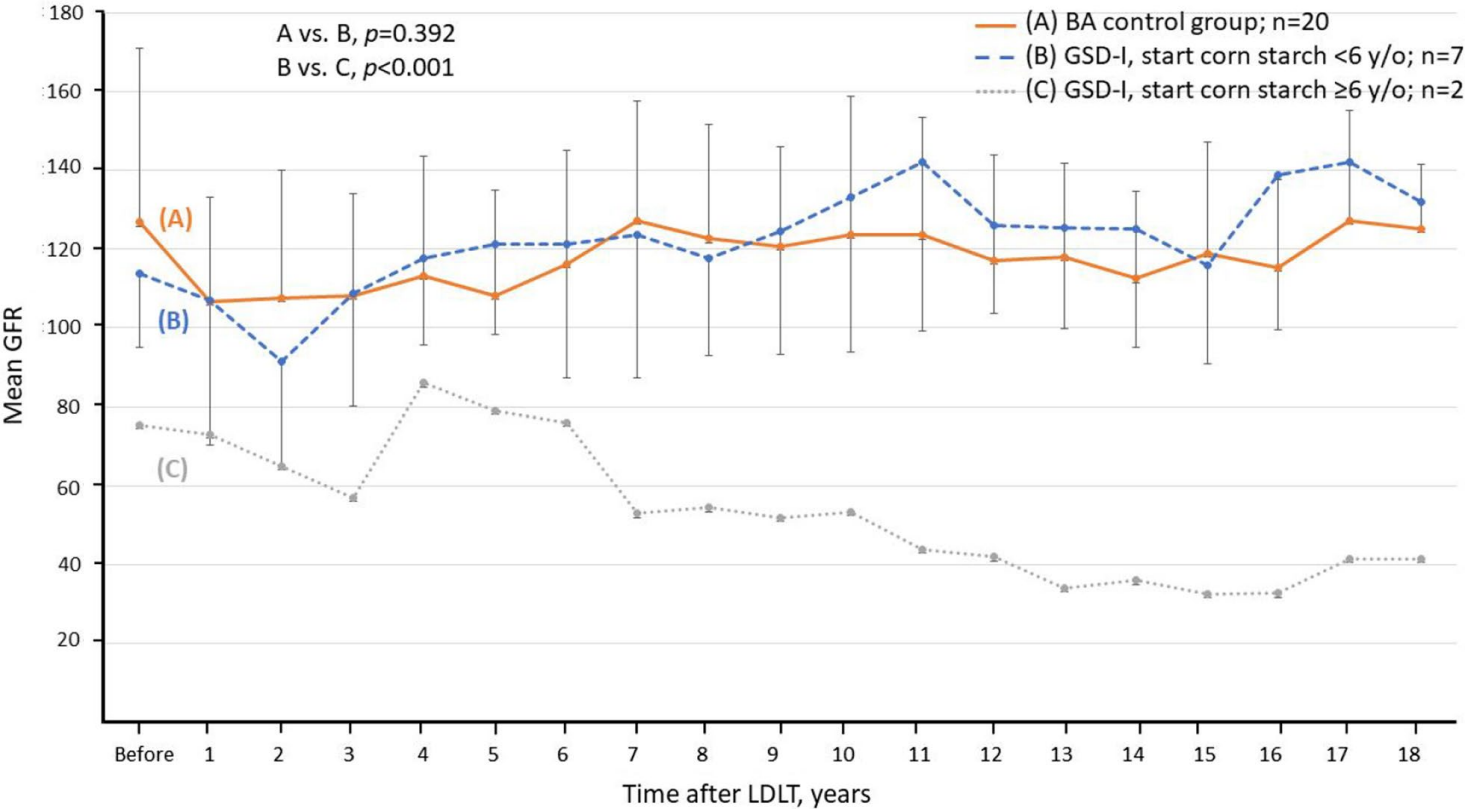
In generalized estimating equation, dynamic change of renal function stratified by glomerular filtration rate (mean GFR; ± 1 standard deviation) in children who underwent LDLT for biliary atresia (group A, n = 20), GSD receiving cornstarch younger than 6-years-old (group B, n = 7) and GSD receiving corn starch older than 6-year-old (group C, n = 2). The renal function was comparable between group A and B (*p* = 0.569), but significant deteriorated renal function in group C versus group B (*p* < 0.001)

We compared the dynamic change in mean eGFR among the GSD-I and BA recipients (Fig. 2). The patients were categorized into three groups: Group A was the BA cohort, group B were GSD-I patients who were started on cornstarch therapy before reaching 6 years of age, and group C were GSD-I patients who were started after the age of 6. The mean eGFR was comparable between A and B (*P* = 0.392), while significant deterioration in renal function in group C when compared with group B.

### Albuminuria

**Fig. 3:**
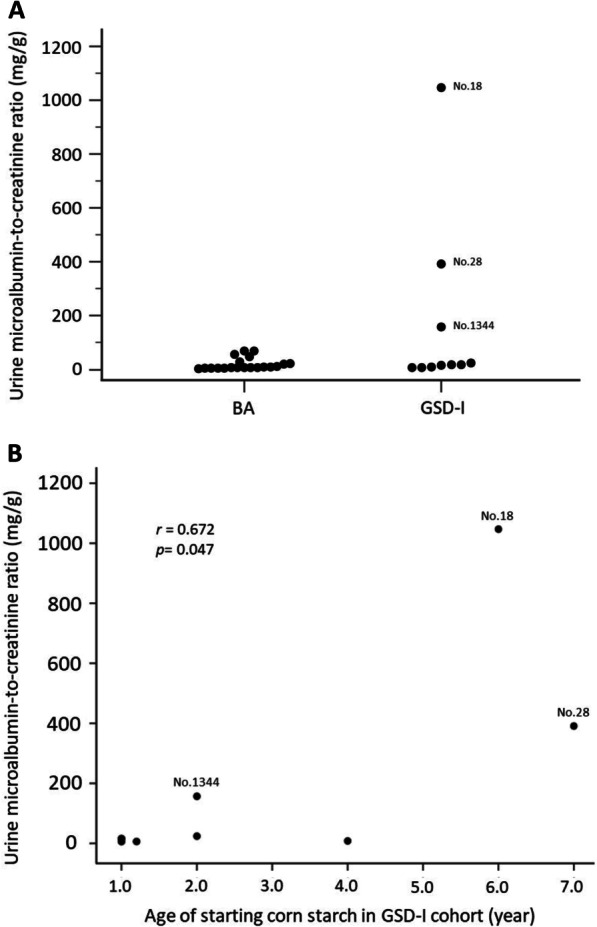
**a** Microalbuminuria in biliary atresia and GSD-I cohorts after LDLT. **b** Relationship between microalbuminuria and age of starting cornstarch in GSD-I cohort

As shown in Table 1 and Fig. 3a, the ACR and incidence of macroalbuminuria (ACR ≥ 300 mg/g) were not significantly different in the GSD and BA groups (median ACR 16.0 vs. 7.2 mg/g, *P* = 0.099; incidence of macroalbuminuria: 22% vs. 0%, *P* = 0.089). But there was a correlation between age of starting cornstarch and microalbuminuria (*r* = 0.672; *p* = 0.047; Fig. 3b). Similarly, according to the division based on the age when cornstarch therapy was initiated in GSD-I patients as mentioned above, group C demonstrated a higher level of ACR (median ACR 719.0 vs. 15.7 mg/g, *P* = 0.040), and a higher prevalence of macroalbuminuria (100% vs. 0%, *P* = 0.030) than group B.

### Renal ultrasonography

**Fig. 4:**
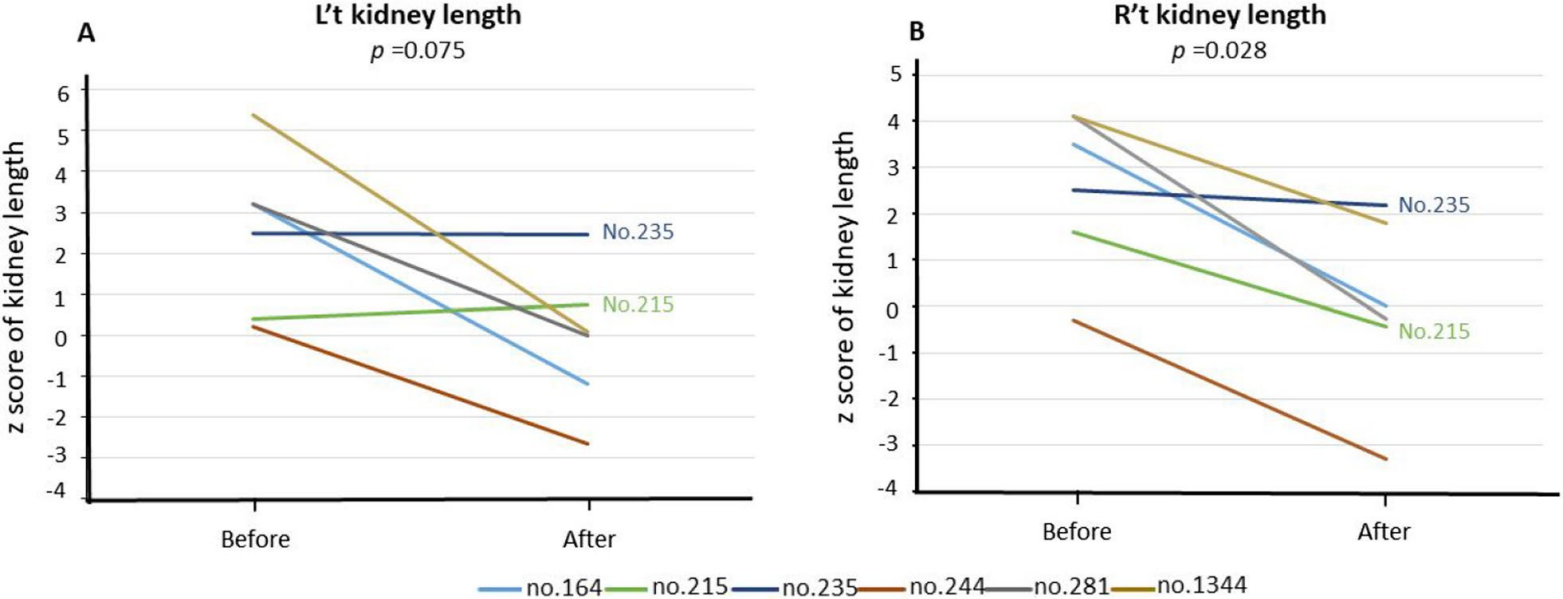
Changes of bilateral kidney length before and after LDLT, expressed by Z scores. **a** Left kidney length, **b** right kidney length

Routine pre-LDLT renal ultrasonography for GSD-I cases was only protocolized in January 2004. Data of GSD-I patients with pre-LDLT ultrasonography (n = 6) was analyzed (Table 3). Regression in kidney size was observed in four patients after LDLT and showed more significant improvement in the right kidney than the left kidney (*P* = 0.028 and 0.075, respectively). For the other two GSD-I recipients, No. 215 and 235, no significant changes in kidney size were observed after LDLT. These changes have been graphically represented in Fig. 4. On the other hand, surveillance renal ultrasonography also revealed findings of new renal cysts (0.8–1.8 cm) in three patients (Nos. 28, 215 and 1344); a calyceal stone in patient No.244; and medullary nephrocalcinosis in patient No. 235.

**Table 3:** The preoperative and postoperative change of eGFR, bilateral kidney length in the sonography, and microalbuminuria

LT No	Age at diagnosis (y)	Age at starting cornstarch (y)	Duration of cornstarch (y)	Age at LT (y)	Follow-up (y)	Post-LT AKI, KDIGO stage	eGFR (ml/min/m^2^)		L kidney length (Z score)		R kidney length (Z score)		Urine microalb/Cr ratio (mg/g)	Adenoma when LT
							Before^#^	After^#^	Before^#^	After^#^	Before^#^	After^#^		
18	4	6	8.4	14.4	22.4	Y, stage 2	58	18	NA	0.6	NA	− 0.4	1047.0	Y
27	1	1	3.4	4.4	21.2	N	127	132	NA	− 0.3	NA	2.2	5.6	N
28	2	7	2.5	9.5	21.2	Y, stage 1	93	53	NA	− 2.1	NA	1.2	391.0	Y
164	1.2	1.2	3.0	4.2	16.6	N	135	111	3.2	− 1.2	3.5	0.0	6.2	N
215	2	2	7.0	9.0	15.4	N	117	144	0.4	0.7	1.6	− 0.4	24.0	N
235	1	1	14.7	15.7	15.0	N	104	97	2.5	2.5	2.5	2.2	15.7	Y
244	1	1	7.8	8.8	14.8	N	98	108	0.2	− 2.7	− 0.3	− 3.3	16.0	N
281	4	4	1.9	5.9	14.0	Y, stage 2	85	175	3.2	0.0	4.1	− 0.3	8.0	N
1344	2	2	11	13	4.1	Y, stage 2	130	128	5.4	0.1	4.1	1.8	157.0	Y

^#^Before means pre-liver transplantation; After means the latest time of follow-up

^*^No. 18 and 28 presented with eGFR < 60 ml/min/m^2^ and microalbuminuria

AKI—acute kidney injury, defined by Kidney Disease Improving Global Outcomes (KDIGO) classification systems; eGFR—estimated glomerular filtration rate; LT—liver transplantation

## Discussion

Whether correction of liver derangement prevents complications of GSD-I remains inconclusive. The liver transplant cases seem to be a good model to explore this possibility. However, such research is quite commonly- limited by the small study populations. In addition, the progression of late complications such as nephropathy may be best observed with long-term surveillance. While LT is theorized to help maintain a healthy metabolic environment, the necessary use of immunosuppressive agents has been a source of inquiry as to whether it compound the risk of developing the sequelae that LT claims to mitigate, that is renal dysfunction.

Here, we have provided a follow-up data of up to 22 years (median follow-up of 15 years), with multiple timepoints of assessment, in 9 GSD-I patients who underwent LDLT in our institution. Our results revealed that the overall renal function, particularly with eGFR and albuminuria as quantitative measures, were not statistically different between the two groups (median eGFR of GSD-I and BA: 111 vs. 123 ml/min/1.73 m^2^, *P* = 0.168; median ACR: 16.0 vs. 7.2 mg/g, *P* = 0.099). Patients with enlarged kidney in GSD-I patients may return to within normal range for age after LDLT. However, a subgroup analysis of the GSD-I cohort showed that patients who initiated cornstarch therapy at or beyond 6 years of age before LDLT had a propensity to develop poorer renal outcomes as measured in terms of albuminuria (*P* = 0.030) and eGFR changes (*P* < 0.001) over time.

Chronic kidney disease is considered a major problem of GSD-I, first noted in 1988 [2]. Worsening renal function was observed in 6 out of 38 GSD-I patients (16%) receiving glucose therapy, with increasing incidence by age, leading to three deaths secondary to renal failure. Even after LT, some patients may progress to CKD. In the literature review by Boers et al., among 80 GSD-I patients undergoing LT, approximately 20% of patients experienced subsequent renal failure [15]. In our cohort of GSD-I, the eGFR remained stabilized or improved after LT among 7 out of 9 patients (78%) over a median follow-up of 15 years. In contrast, the other two patients (22%) presented with eGFR < 60 ml/min/1.73 m^2^ and marked albuminuria more than 20 years after LT. Given its relative rarity, limited numbers of GSD-I recipients could be included in this study, inadequate to conclude with statistically significant predictive variables for post-LT renal outcome and prognosis. Nonetheless, through observational analysis of collected long-term retrospective data, divergent trends between the cornstarch therapy initiation groups < 6-years-old and ≥ 6-years-old can be deduced (Tables 2 and 3; Fig. 2); these suggest a possible renal protective role from progression to complicating sequelae. Despite the long-term follow-up of this study cohort, it may still be more prudent to defer any definitive conclusions regarding the development of renal dysfunction and continue observing them prospectively, as renal involvement has been typically observed to progress during adulthood among GSD type 1 patients [24]. Additionally, deterioration of renal function in patient No. 18 may be explained by the natural course of kidney disease as the renal function was already compromised before LT. The declining renal function after LT in patient No. 28 patient was less clear, and his pre-LT renal function appeared normal; however, it deteriorated gradually after LT.

For GSD-I patients receiving cornstarch therapy without LT, optimization of metabolic control with normal level of blood lactate, serum lipids, and uric acid may delay or prevent kidney damage [6, 10]. A previous report demonstrated that albuminuria was retrospectively observed in patients who started cornstarch therapy at a later age (9.3 ± 3.8 years, n = 8); patients who did not develop albuminuria started therapy younger (5.7 ± 4.1 years, n = 18) [25]. Our results were consistent with these findings; two patients (No. 18 and 28) with late age of starting cornstarch therapy (≥ 6-year-old) exhibited macroalbuminuria and decreased eGFR compared to other patients. Although this hypothesis was based on observations, it suggests that the age of starting cornstarch therapy in GSD-I pre-school patients may be critical for renal outcomes. Microalbuminuria is usually the first sign of glomerular damage in GSD-I patients, followed by proteinuria, systemic arterial hypertension, and renal failure [9, 26]. The 2002 European Study on GSD-I reported that the prevalence of microalbuminuria (ACR 2.5–20) and proteinuria (ACR > 20) was 31% and 13%, respectively, in GSD-I patients under dietary control [27]. The incidence of both increased with age, such that 100% of patients over 25 years of age developed microalbuminuria, and more than half concurrently presenting with proteinuria [27]. However, in our study, two of GSD-I patients (No. 27 and No. 235) who were over 25 years of age at last follow-up, did not presented with microalbuminuria (Table 3). These results imply that LT may preserve kidney function in GSD-I patients. Interestingly, post-LT arterial hypertension was also diagnosed in the two GSD-I patients with macroalbuminuria (No. 18 and 28), in coinciding with their diagnosis of renal failure (Table 2). Therefore, monitoring urine protein level and blood pressure may alert clinicians as to the onset or progression of renal dysfunction.

When analyzing the factors affecting the long-term renal function in GSD-I patients, AKI occurrence was not associated with CKD in our results. Although the recent evidence pointed that pediatric AKI attributed to several adverse long-term consequences, including proteinuria, hypertension, reduced eGFR, and CKD [28, 29]. In our cohort, however, post-LT AKI was not associated with inferior renal outcomes in GSD and BA groups. The reasons might come from that LT surgery was the main etiology of AKI rather than sepsis and the full recovery of renal function in our patients, both of which were proved to be good indicators of AKI prognosis [28–30].

Liver transplantation provides a healthy liver graft to maintain a normal metabolic environment, however, the side effect of immunosuppression poses a risk of CNI related nephrotoxicity [31, 32]. Usually, immunosuppression is tapered gradually after transplant surgery when the risk of rejection decreases, and then GFR increases accordingly. Studies by Berg et al. and Arora-Gupta et al. reported that GFR was reduced during the first year after LT [31, 32], which is consistent with our results (Fig. 2). With reduction in immunosuppression therapy to a lower dosage one year after LT, eGFR increased and subsequently stabilized in most of our patients (Fig. 2). The immunosuppression regimen was switched from CNI to mTOR in patients Nos. 18 and 28 due to elevated SCr and in patient No. 1344 due to post-transplant lymphoproliferative disorder of CNI. Progression of ESRD was unmanageable for patient No. 18 but reversible for No. 28 (Table 2), which might be explained by pre-existing renal dysfunction in patient No. 18 before LT.

Literature estimates that 46–70% of GSD-I patients may develop nephromegaly with age-adjusted lengths that exceed 2 SDs [6, 26]. In our study, four of six patients (67%; No. 164, 235, 281, and 1344) demonstrated enlarged kidneys (exceeding 2 SDs) before LDLT (Table 3). As children grow up, the kidney is expected to physiologically enlarge. However, when the kidney length was translated into z score and adjusted for age, we observed regression in the kidney length to within acceptable length-for-age in three of four GSD-I patients (75%; no. 164, 281, and 1344), and non-progression in the fourth patient (no. 235), (Table 3; Fig. 4). From these observations, LT did reverse nephromegaly, and this may be attributable to the normalized metabolic environment with resolution of dyslipidemia, which was regarded as one of risk factors for nephromegaly [6].

This study has likewise been consistent in terms of the role of LT in reversing failure-to-thrive, improvement of hepatic function, and reversal of metabolic dysfunction in GSD-I patients [7, 33]. Furthermore, neutropenia in the GSD-Ib patient improved after LDLT, implying that correction of liver derangement may have potential benefit with concomitant immune disorder [14].

Gene therapy, gene editing, and mRNA therapy are new potential strategies for treating genetic diseases [34]. The liver is an important and common target for such strategies [35]. Our data indicated that correction of liver derangement by liver targeting may be a critical strategy in preventing complications related to other organs in GSD-I patients.

## Limitations of the study

This study has several limitations. First, we are unable to specifically identify confounding factors associated with renal outcome from our available data, primarily due to early manually recorded information that could not be retrieved from our hospital's previous manual (non-electronic) medical records, as well as missing parameters from transferred medical records of patients from other hospitals. Second, the study was conducted at a single medical center, and postoperative prognosis may vary among different hospitals by virtue of differences in management. Third, the GFR was calculated using the updated Schwartz formula for children and the MDRD formula for adults, both of which may overestimate or underestimate the true GFR. However, we used the formula in both groups for comparability to minimize bias. Fourth, we may underestimate the incidence of AKI because only SCr values were used as the main criterion in the definition of AKI and urinary output was eliminated from the equation. Finally, the study recruited only 9 GSD-I and 20 BA patients. Therefore, to surpass the limitations, a larger, prospective, randomized controlled trial would be ideal.

## Conclusions

Our clinical data demonstrated that post-LT renal function was well preserved in most GSD-I patients. LT cannot reverse the preoperative renal dysfunction but may prevent or slow the progression of albuminuria and CKD. The timepoint of starting cornstarch therapy in GSD-I patients of pre-school age may be critical for long-term renal function. Early initiation of the treatment results in a good renal prognosis.

## Supplementary Information

**Additional file 1: Supplementary Table 1.** Primers for the exon sequencing of *G6PC* and *SLC37A4* gene.

## Data Availability

The dataset supporting the conclusions of this article are included within the article and files. The dataset used during the current study are available from the corresponding author on reasonable request.

## Abbreviations

AKI: Acute kidney injury
AST: Aspartate transaminase
BA: Biliary atresia
BH: Body height
Bil: Total bilirubin
BMI: Body mass index
BW: Body weight
Chol: Total cholesterol
CKD: Chronic kidney disease
CM: Centimeters
CNI: Calcineurin inhibitor
CT: Computed tomography
CyA: Cyclosporin A
eGFR: Estimated glomerular filtration rate
ER: Endoplasmic reticulum
ESRD: End-stage renal disease
FK: Tacrolimus
GRWR: Graft to recipient weight ratio
GSD: Glycogen storage disease
G6P: Glucose 6-phosphate
HTN: Hypertension
KDIGO: Kidney Disease Improving Global Outcome
LDLT: Living donor liver transplantation
LT: Liver transplantation
MDRD: Modification of Diet in Renal Disease
mTOR: Mammalian target of rapamycin
PCR: Polymerase chain reaction
PELD: Pediatric end-stage liver disease
SCr: Serum creatinine
SDs: Standard deviations
TG: Triglyceride
UA: Uric acid
