# Supplementary material for: Modifiable factors affecting renal preservation in type I glycogen storage disease after liver transplantation: a single-center propensity-match cohort study
Source: Orphanet J Rare Dis. 2021 Oct 11;16:423. doi: 10.1186/s13023-021-02026-6 (PMC8507322; doi:10.1186/s13023-021-02026-6)
Supplement: Supplementary file 1 — Additional file 1: Supplementary Table 1. Primers for the exon sequencing of G6PC and SLC37A4 gene. [file 13023_2021_2026_MOESM1_ESM.docx]

Supplementary Table 1. Primers for the exon sequencing of *G6PC* and *SLC37A4* gene

| **Name** | **PCR product size (bp)** | **Sequence** |
| --- | --- | --- |
| G6PC_Ex1_F | 375 | ATAGCAGAGCAATCACCACC |
| G6PC_Ex1_R |  | ACAGACATTGCGAGAGCGAA |
| G6PC_Ex2_F | 192 | GCATTCATTCAGTAACCC |
| G6PC_Ex2_R |  | TCCACTCAGCTTCTGTCT |
| G6PC_Ex3_F | 209 | CACCTTTACTCCATTCTC |
| G6PC_Ex3_R |  | GTGGTGTGTCAGCTACA |
| G6PC_Ex4_F | 259 | GCCAGGCTCCAACATTT |
| G6PC_Ex4_R |  | GGAGAGAAACGGAATGG |
| G6PC_Ex5_F | 647 | CTTCCTATCTCTCACAG |
| G6PC_Ex5_R |  | TCACTTGCTCCAAATACC |
| SLC37A4_Ex1_F | 478 | TGTTCGGTGTGTTTTGTAGAGG |
| SLC37A4_Ex1_R |  | CGATAGAAAGGGGTCTCGTG |
| SLC37A4_Ex2a_F | 754 | TTCAGGTAGGAGGCGGTATG |
| SLC37A4_Ex2a_R |  | ACACAGAAAGCCCCGCTAAG |
| SLC37A4_Ex2b_F | 558 | AGTAGCGTGTCCTGGGTAGC |
| SLC37A4_Ex2b_R |  | TTTCCTACAGTTGCCCCTTG |
| SLC37A4_Ex3_F | 602 | AAGCTGGTTTCACAGGCAAG |
| SLC37A4_Ex3_R |  | AGACAATCCACCCACACCAC |
| SLC37A4_Ex4_F | 565 | CAGGCTCCTCCTGTTCCTCT |
| SLC37A4_Ex4_R |  | AGTTTCCTCCTCTGCCACCT |
| SLC37A4_Ex5-6_F | 597 | GCCAGCTCCTATTGCTTGTC |
| SLC37A4_Ex5-6_R |  | GGGATGGATTTGTGTTTGCT |
| SLC37A4_Ex7_F | 491 | TCCCTGGTCCTGTTTCTCAT |
| SLC37A4_Ex7_R |  | GGCCATAGCTGCCTGAGTAG |
| SLC37A4_Ex8_F | 445 | GGCCTTGCTTCCATTTATCC |
| SLC37A4_Ex8_R |  | TTCCACAACCGTAGGAAAGC |
| SLC37A4_Ex9_F | 417 | CAAAGACCCGTCAGCAGAG |
| SLC37A4_Ex9_R |  | GGAGCACAGGGAAGAAAAGA |
| SLC37A4_Ex10_F | 410 | CCAATCTTTTCTTCGCCTCTC |
| SLC37A4_Ex10_R |  | CTTCCACTCCCCAACACAAC |
| SLC37A4_Ex11_F | 522 | TTTGCTCAATGTGGCATCTC |
| SLC37A4_Ex11_R |  | CAGAAGGCTGTGCTCCAACT |
| SLC37A4_Ex12a_F | 605 | CCCACCTGTGCTTTGCAT |
| SLC37A4_Ex12a_R |  | GCAGGGATAGCCTCACTTCA |
| SLC37A4_Ex12b_F | 572 | CCATTTCTGCGCCTTTTCT |
| SLC37A4_Ex12b_R |  | ACACTTCAGCTTCCCCATTC |
